# Seasonal distribution of human-to-human pathogens in airborne PM_2.5_ and their potential high-risk ARGs

**DOI:** 10.3389/fmicb.2024.1422637

**Published:** 2024-07-04

**Authors:** Zhiwei Zuo, Yuanyuan Pan, Xueyun Huang, Tao Yuan, Cheng Liu, Xihong Cai, Zhongji Xu

**Affiliations:** ^1^Jiangxi Provincial Key Laboratory of Genesis and Remediation of Groundwater Pollution, East China University of Technology, Nanchang, China; ^2^Jiangxi Center for Patriotic Health and Health Promotion, Nanchang, China

**Keywords:** PM_2.5_, human-to-human pathogens, seasonal variation, hub pathogens, antibiotic resistance genes (ARGs)

## Abstract

Airborne microorganisms, an emerging global health threat, have attracted extensive studies. However, few attentions have been paid to the seasonal distribution of airborne pathogens, in particular their associations with antibiotic resistance genes (ARGs). To this end, two-week daily PM_2.5_ samples were consecutively collected from Nanchang in four seasons, and the human-to-human pathogens were screened based on high-throughput sequencing. The results showed that there were 20 pathogenic taxa in PM_2.5_ in Nanchang, and the highest relative abundance of pathogens was observed in winter (5.84%), followed by summer (3.51%), autumn (2.66%), and spring (1.80%). Although more than half of pathogenic taxa were shared by the four seasons, the analysis of similarities showed that pathogenic community was shaped by season (*r* = 0.16, *p* < 0.01). Co-occurrence network analysis disclosed significant interactions among pathogens in each season. Moreover, some dominant pathogens such as *Plesiomonas shigelloides*, *Bacteroides fragilis*, and *Escherichia-Shigella* were hub pathogens. In addition, PICRUSt2 predicted that there were 35 high-risk ARG subtypes in PM_2.5_, and the pathogens had strongly positive correlations with these ARGs. Even some pathogens like *Plesiomonas shigelloides*, *Bacteroides fragilis*, *Aeromonas*, *Citrobacter*, may be multi-drug resistant pathogens, including beta-lactam, aminoglycosides, chloramphenicol and multi-drug resistances, etc. Both air pollutants and meteorological conditions contributed to the seasonal variation of airborne pathogenic bacteria (*r* = 0.15, *p* < 0.01), especially CO, O_3_, PM_2.5_, temperature and relative humidity. This study furthers our understanding of airborne pathogens and highlights their associations with ARGs.

## 1 Introduction

Bacteria, the main component of atmospheric particulate matter, are prevalent in the near-surface atmosphere at concentrations of about 10^4^–10^8^ cells/m^3^ (Bowers et al., 2011; Yan et al., 2022). Airborne bacteria can not only be involved in the formation of clouds and rains as cloud/ice condensation nuclei, affecting the atmospheric physicochemical processes (Wei et al., 2017; Tang et al., 2022), but also pose a threat to human health. Because some human pathogens were detected among airborne microorganisms, accounting for 0.35–26.72% of total airborne bacteria (Du et al., 2018; Fan et al., 2019). Furthermore, unlike drinking water and food, atmospheric particulate matters are generally untreated and directly contact with the human skins or are inhaled by human. Therefore, airborne pathogens attaching on particles may lead to skin irritation or inflammation (Callewaert et al., 2020), respiratory diseases (Xue et al., 2020), cardiovascular disease (Riggs et al., 2018), and so on.

Although numerous studies were conducted about atmospheric bacteria (Files et al., 2020; Ruiz-Gil et al., 2020), systematic studies on airborne pathogenic bacteria were scarce. Moreover, the abundance and composition of pathogenic bacteria varied greatly in the existing reports. For example, Du et al. (2018) have found that pathogenic bacteria in Beijing occupied 0.24–0.75% of the total bacteria, including *Streptococcus*, *Prevotella*, *Erysipelothrix*, *Rickettsia*, and *Enterobacter*, while Fan et al. (2019) have reported that the relative abundance of airborne pathogens in Beijing was 1.82–14.50%, containing 22 pathogenic genera such as *Bacillus*, *Pseudomonas, Acinetobacter*, and *Clostridium*. In particular, Lu et al. (2018) have reported that airborne pathogens in Xi’an accounted for higher than 20% of total bacterial community whether in clean or hazy days. This bias may be contributed by the different geophysical locations and sampling time. In additional, most of these potential pathogens were counted at the genus level, where some species involved may not be human pathogens. More accurate statistics are necessary.

Antibiotic resistance genes (ARGs) in atmospheric particulate matter have received widespread attention in recent years (Li et al., 2018; Yan et al., 2022; Ma et al., 2023). Several to hundreds of ARG subtypes have been detected in the atmosphere, with an absolute abundance about 10^4^–10^5^ copies/m^3^. Some high-risk ARG subtypes, such as *sul*1, *tet*M, *bla*TEM-1, and so on. If these ARGs are carried by human pathogens, once infected by human, they will greatly reduce the effectiveness of antibiotics, increase the cost and duration of treatment, and even lead to death (Zeng et al., 2019). However, the relationship between these ARGs and pathogens in the air remained limited.

Herein, seasonal distribution of human-to-human pathogens in airborne PM_2.5_ and their potential-carried ARGs were explored. Particle samples in different seasons were collected from Nanchang, the capital of Jiangxi Province, where PM_2.5_ are still one of the primary pollutants in the air. Moreover, our previous study found that several potential pathogens like *Pseudomonas*, *Clostridium*, and *Bacillus*, were detected in the airborne bacterial community in Nanchang (Pan et al., 2022). Zhu et al. (2021) also found that more than 100 ARG subtypes in the snow samples collected from Nanchang. This study aimed to systematically investigate seasonal variation of pathogenic abundance and community composition, find out the key pathogens and potential ARGs hosts, and finally explore their associations with environmental factors. Overall, this study advances our understanding on seasonal distribution of airborne pathogens and highlights the health risk of ARGs potential-carried by airborne pathogens.

## 2 Materials and methods

### 2.1 Sample collection

PM_2.5_ samples were collected on the roof of the Earth Science Building in East China University of Technology (28.72°N, 115.82°E). There were no obvious emission sources around the sampling site, but it was surrounded by residential areas. A high-flow sampler (KC-1000, Qingdao Electrical Equipment Co., Ltd., China) equipped with a PM_2.5_ inlet was used at a flow rate of 1 m^3^/min and 23.5 h for each sample. The daily samples were continuously collected for 2 weeks in each season, and the detailed sampling periods were Apr 30, 2019 – May 13, 2019 (14 samples, spring), Jan 18, 2020 – Jan 30, 2020 (14 samples, winter), Jul 1, 2020 – Jul 15, 2020 (15 samples, summer), and Oct 13, 2020 – Oct 26, 2020 (14 samples, autumn), respectively. To avoid contamination, the sampling filter membrane (8 in × 10 in) was sterilized in a muffle furnace at 450°C for 4 h before use. The inlet was scrubbed using 75% alcohol. A total of 57 samples were collected and stored in a refrigerator at −20°C.

The concentrations of air pollutants (SO_2_, NO_2_, O_3_, CO, PM_2.5_ and PM_10_) and meteorological data [temperature (T), relative humidity (RH), wind speed (WS) and precipitation] were collected from the environmental monitoring station nearest to the sampling point, and integrated according to the actual sampling time.

### 2.2 DNA extraction and sequencing

The DNA extraction method was according to our previous study (Pan et al., 2022, 2024). The filter membrane was firstly ground into powder with agate, and then DNA was extracted with DNeasy PowerSoil Pro Kit (Qiagen, United States) according to the instruction. The extracted DNA was sent to Guangzhou Genedenovo Company for sequencing. The V3–V4 region of 16S rRNA gene was amplified using the specific primers (341F: CCTACGGGNGGCWGCAG; 806R: GGACTACHVGGGTATCT AAT) with barcodes. After purification, the PCR products were quantified and equimolar pooled for sequencing on the Novaseq 6,000 platform. The raw data were uploaded in FASTA format under the SRA database in NCBI [Accession numbers: PRJNA867124 (summer, autumn, winter) and PRJNA867037 (spring)]. The Bioproject ID, Biosample ID and sample name for each season were provided in Supplementary Table S1.

### 2.3 Sequence analysis

After sequencing, the raw data were filtered with FASTP to obtain clean reads. Clean reads were then assembled into tags and denoised with FLASH (1.2.11) to obtain effective tags (Magoč and Salzberg, 2011). Next, effective tags were clustered according to 97% similarity with UPARSE (9.2.64) to obtain OTUs (Edgar, 2013). Chimeras were removed. The valid sequence with the highest abundance in each cluster was selected as the representative OTU sequence, which was annotated according to the SILVA database with a confidence threshold of 0.8 (Pruesse et al., 2007). All samples were resampled according to the lowest number of tags. Finally, an OTU table with relative abundance could be obtained. Bacterial community composition was then calculated for each season. The rarefaction curve was made and reached a plateau (Supplementary Figure S1), suggesting that the employed sequencing depth have captured the full diversity of bacterial community.

### 2.4 Screening of human-to-human pathogens and ARGs prediction

Pathogens in each season were screened according to Human-to-Human Pathogens Database published by^1^ National Health Commission of the People’s Republic of China (2023), where clearly indicated the pathogenic genera or species. ARGs were screened according to the method of Jiang et al. (2022). Firstly, the bacterial function profiles were predicted with PICRUSt2, and we obtained a table of functional genes including gene description and gene counts known as KOs. Secondly, we downloaded the list of ARGs from the KEGG database^2^. Thirdly, we intersected the KO table and ARGs list to obtain the potential ARGs composition. We focused on the high risk ARGs, including Rank I and Rank II ARGs as reported by Zhang A. et al. (2021). The average nearest sequenced taxon index (NSTI) used to evaluate the accuracy of PICRUSt2 results was 0.08 (lower than 0.2), suggesting that the PICRUSt2 results were reliable.

### 2.5 Statistic analysis

Venn diagram was used to show the shared pathogens among four seasons. Student *t*-test was applied to evaluate the differences in the total relative abundance of pathogenic bacteria between two seasons, and analysis of similarity (ANOSIM) based on Bray–Curtis distance was performed to assess the similarity of pathogen composition between seasons using R software. Co-occurrence network based on Spearman’s correlation (*p* < 0.05) was performed using Gephi software (0.9.2) to explore the interaction of pathogen and pathogen and then find out the key pathogens (the number of edges occupying more than 20% of the total edges). Likewise, the potential ARG hosts were also mined based on the significantly positive correlations between the relative abundance of pathogens and gene counts of ARGs. Spearman correlation test was performed using R software. Additionally, mantel test, redundancy analysis (RDA) and partial RDA (pRDA) were used to assess the contribution of environmental factors (air pollutants and meteorological conditions) to variation of airborne pathogens using R software. Figures were plotted using OringinLab 2018 and Omicshare platform.

## 3 Results

### 3.1 Diversity of airborne human-to-human pathogens

**Figure 1:**
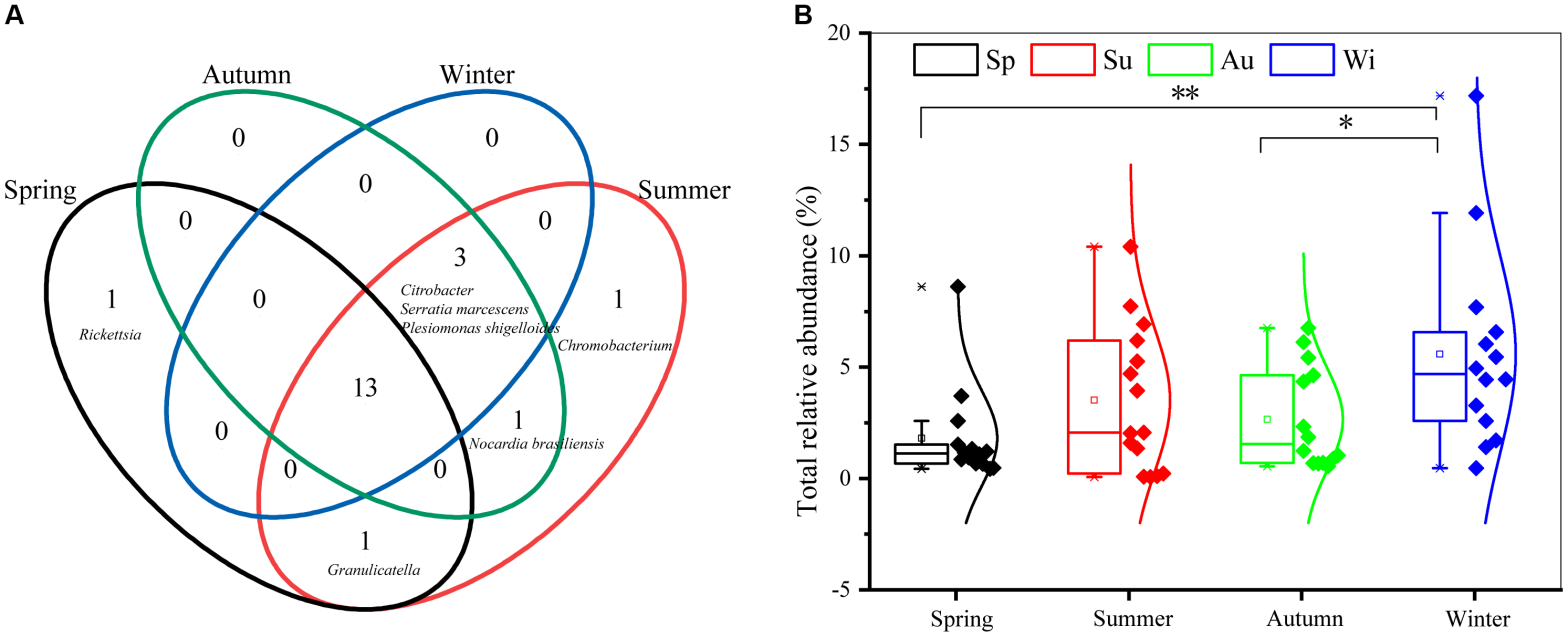
Comparison of pathogenic diversity in different seasons. **(A)** Venn diagram to show the number of shared pathogens among four seasons. **(B)** Statistical comparisons of the total relative abundance of pathogens between seasons. The upper line of the box plot is the maximum value, and the center line is the median, and the lower line is the minimum. The square dot is the mean relative abundance. The diamond dot represents samples. * and ** indicate the significant differences between two seasons with *p* < 0.05 and *p* < 0.01, respectively.

There are 20 human-to-human pathogenic taxa (genera and species) in PM_2.5_ in Nanchang. The number of pathogenic taxa in each season followed the pattern, summer (19), autumn (17), winter (16), spring (15) (Figure 1A). However, most of these pathogens (13) were shared by four seasons, including species from *Prevotella*, *Escherichia-Shigella*, *Streptococcus*, and so on. Notably, *Rickettsia* was only observed in spring, and *Chromobacterium* was a summer-specific pathogenic genus.

The total relative abundance of pathogens ranged from 0.07 to 12.21% in all samples, where the shared pathogens by four seasons accounted for 88.25–99.61% in each season. The average of total abundance was highest in winter (5.84% ± 4.44%), followed by summer (3.51% ± 3.25%), autumn (2.66% ± 2.29%), and spring (1.80% ± 2.15%) (Figure 1B). *T*-test showed the total relative abundance of pathogens in winter was obviously higher than that in spring and autumn.

### 3.2 Community composition of airborne human-to-human pathogens

**Figure 2:**
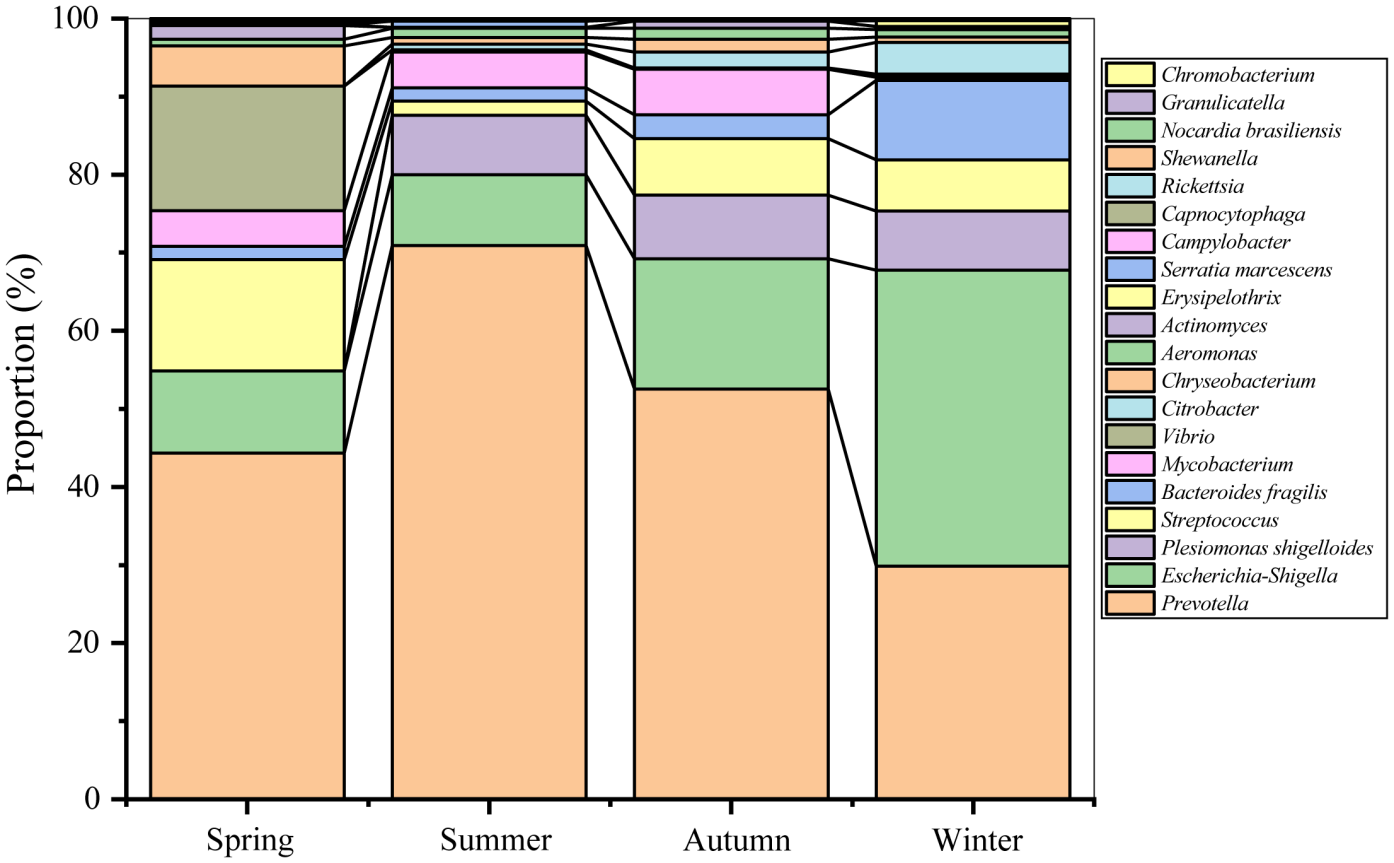
Diagram of pathogen composition based on the proportion of each pathogen in all pathogens in each season.

As Figure 2 shown, *Prevotella* (1.59%), *Escherichia-Shigella* (0.77%), *Plesiomonas shigelloides* (0.23%), *Streptococcus* (0.22%), *Bacteroides fragilis* (0.18%), and *Mycobacterium* (0.11%), were the dominant pathogens (relative abundance >0.10%) in Nanchang. Among these pathogens, *Prevotella* and *Escherichia-Shigella* were found to be dominant in all seasons, and *Mycobacterium* and *Plesiomonas shigelloides* were shared by three seasons. However, *Bacteroides fragilis* occupied higher in winter. Additionally, *Vibrio* and *Citrobacter* were enriched in spring and winter, respectively.

ANOSIM test showed that distinct differences were observed for pathogenic community composition among four seasons (*r* = 0.16, *p* = 0.00). Except between summer and autumn/winter, significant differences in pathogenic communities were observed between the other seasons (Table 1). In details, *Actinomyces*, *Campylobacter*, *Chryseobacterium*, *Vibrio*, and *Rickettsia*, were enriched in spring, while the relative abundances of *Plesiomonas shigelloides* and *Prevotella* were lower than that in other seasons (Figure 2 and Supplementary Figure S2). Similarly, *Granulicatella*, *Capnocytophaga*, *Prevotella*, *Serratia marcescens*, *Nocardia brasiliensis*, *Chromobacterium* were abundant in summer, while *Actinomyces* and *Streptococcus* were less abundant than other seasons. *Shewanella*, *Escherichia-Shigella*, *Bacteroides fragilis*, *Citrobacter*, and *Erysipelothrix* were enriched in winter, while a lower relative abundance was for *Mycobacterium* compared with other seasons. There was no enrichment of pathogens in autumn, whereas *Campylobacter* was noticeably less abundant than other seasons.

**Table 1:** Comparison of pathogenic community between seasons by ANOSIM test.

Groups	*r*	*p*
Spring vs. Summer	0.241	0.002
Spring vs. Autumn	0.131	0.029
Spring vs. Winter	0.378	0.000
Summer vs. Autumn	0.025	0.216
Summer vs. Winter	0.062	0.069
Autumn vs. Winter	0.144	0.022

### 3.3 Interactions between airborne pathogens in different seasons

**Figure 3:**
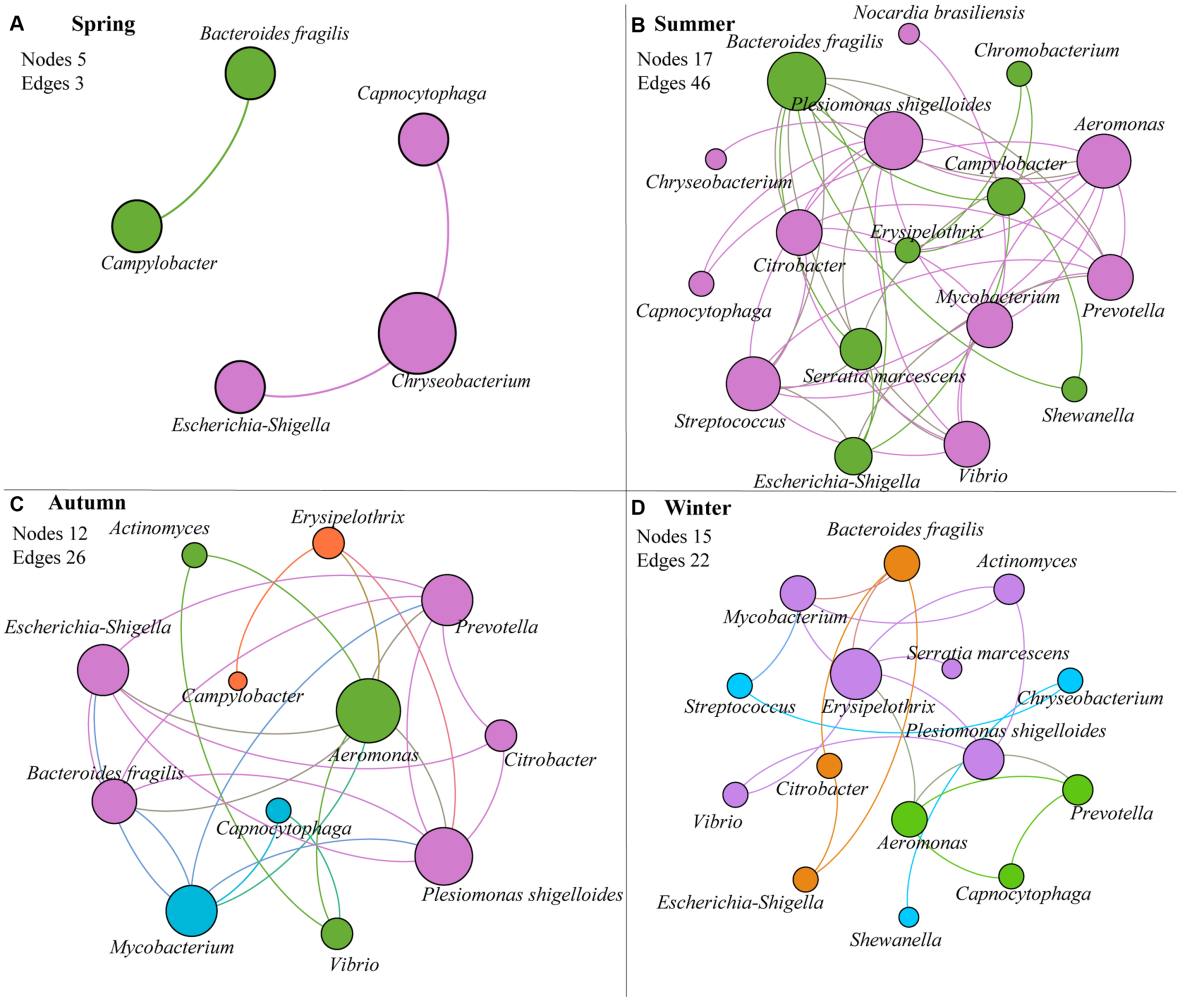
Co-occurrence interaction network between pathogens based on Spearman’s correlation (*p* < 0.05) of their relative abundances in each season. **(A)** spring; **(B)** summer; **(C)** autumn; **(D)** winter. Node size is proportional to node degree; edges represent interactions between nodes. The nodes (pathogens) with the same color are from the same modularity class.

The interactions between human-to-human pathogens were evaluated for each season. Except spring, most of pathogens (12–17) in the other seasons (in particular summer) were closely related (Figure 3, Table 2, and Supplementary Table S2). The total number of edges in summer, autumn, winter, was 46, 26, and 22, respectively. Moreover, *Plesiomonas shigelloides* and *Bacteroides fragilis* played the core role in the interaction network in summer. *Aeromonas*, *Plesiomonas shigelloides*, *Mycobacterium*, *Escherichia-Shigella*, and *Prevotella* were the hub pathogens in autumn, and *Erysipelothrix* and *Plesiomonas shigelloides* was also the key pathogen in winter. Among these hub pathogens, the relative abundances of some bacteria taxa like *Bacteroides fragilis*, *Aeromonas*, and *Erysipelothrix* was less than 0.10%.

**Table 2:** Edge number and relative abundance of each node in pathogen-pathogen co-occurrence network.

Nodes	Spring		Summer		Autumn		Winter	
	Edges	Abundance^a^ (%)	Edges	Abundance (%)	Edges	Abundance (%)	Edges	Abundance (%)
*Bacteroides fragilis*	1	0.031	**10** ^**b**^	**0.059**	5	0.081	4	0.568
*Plesiomonas shigelloides*	0	0.000	**10**	**0.267**	**7**	**0.217**	**5**	**0.421**
*Aeromonas*	0	0.015	9	0.041	**8**	**0.037**	4	0.053
*Streptococcus*	0	0.257	9	0.065	0	0.193	2	0.366
*Mycobacterium*	0	0.083	7	0.162	**6**	**0.155**	4	0.024
*Vibrio*	0	0.288	7	0.009	3	0.004	2	0.022
*Citrobacter*	0	0.000	7	0.027	3	0.055	2	0.228
*Prevotella*	0	0.800	7	2.491	**6**	**1.398**	3	1.665
*Serratia marcescens*	0	0.000	6	0.028	0	0.006	1	0.006
*Campylobacter*	1	0.006	5	0.004	1	0.000	0	0.005
*Escherichia-Shigella*	1	0.189	5	0.318	**6**	**0.443**	2	2.117
*Chromobacterium*	0	0.000	2	0.000	0	0.000	0	0.000
*Erysipelothrix*	0	0.002	2	0.000	3	0.000	**7**	**0.039**
*Shewanella*	0	0.001	2	0.001	0	0.002	1	0.003
*Capnocytophaga*	1	0.001	2	0.003	2	0.000	2	0.003
*Nocardia brasiliensis*	0	0.000	1	0.003	0	0.000	0	0.000
*Chryseobacterium*	2	0.093	1	0.029	0	0.044	2	0.037
*Actinomyces*	0	0.032	0	0.004	2	0.025	3	0.023

^a^Abundance represents the relative abundance in all bacteria. ^b^Bold number corresponds to the key pathogen taxa.

### 3.4 Potential ARGs carried by airborne pathogens

**Figure 4:**
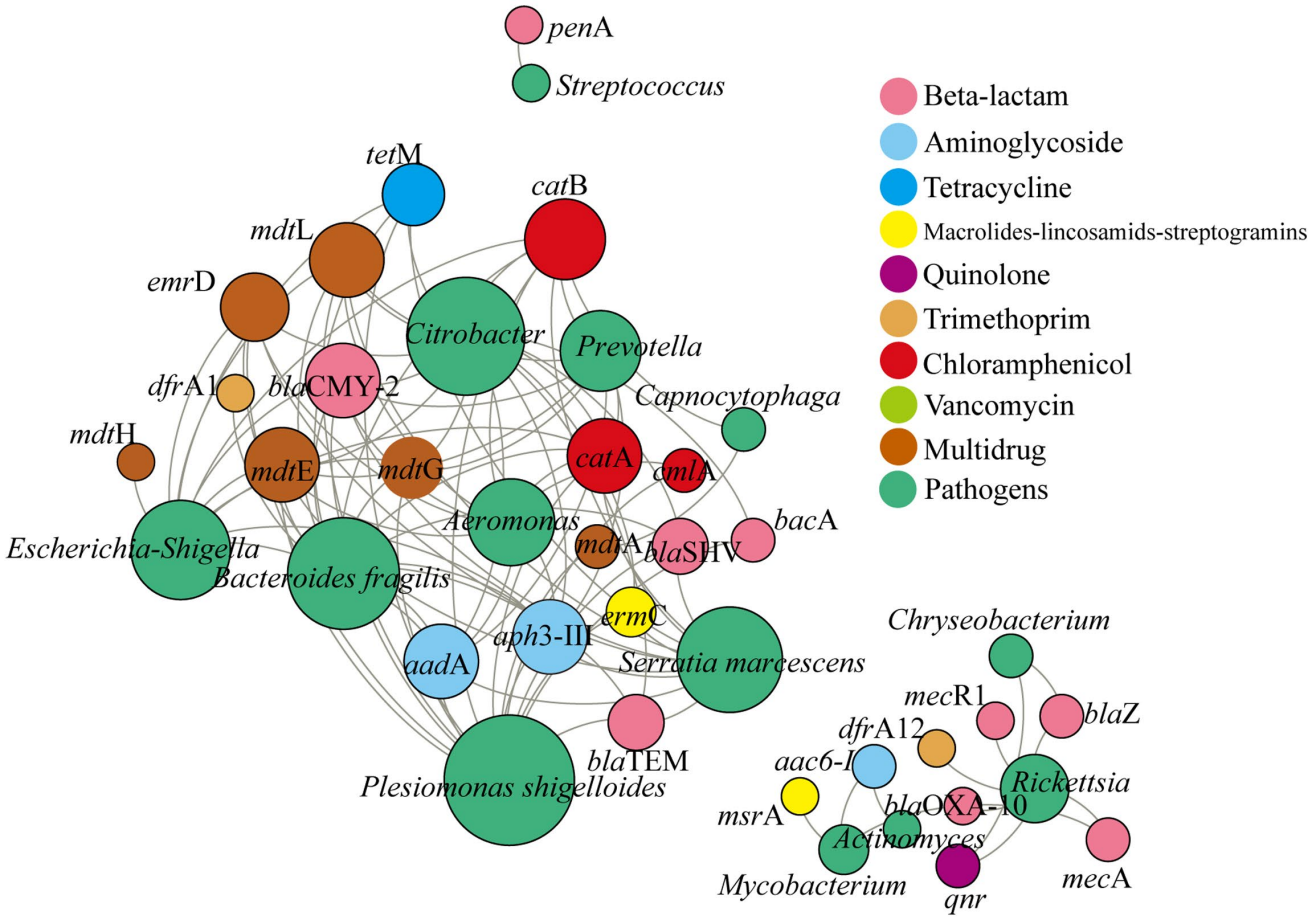
Co-occurrence network analysis between pathogens and potential ARG subtypes based on Spearman correlation between the relative abundance of pathogens and gene counts of ARGs subtypes (predicted by PICRUSt). Only the positive and significant correlations between pathogens and ARGs were displayed. The size of each node is proportional the degree, and the color represents the antibiotic types and pathogens.

There were 35 high-risk ARG subtypes (Rank I and Rank II) predicted in the atmosphere in Nanchang. The predominant ARGs (gene counts >5,000) included *bac*A (*β*-lactam), *cat*A (chloramphenicol), *mdt*A (multidrug), *tet*M (tetracycline), *mep*A (multidrug), and *van*Y (vancomycin). *T*-test showed that there were significant differences on the total abundance of ARGs between seasons, except between winter and summer, and the total abundance of ARGs followed the pattern, summer > winter > autumn > spring. Furthermore, human-to-human pathogens were significantly associated with these ARGs, and each pathogen carried an average of 7.54 ARG subtypes (Figure 4). The above core pathogens like *Plesiomonas shigelloides*, *Escherichia-Shigella*, and *Bacteroides fragilis* were positively related with more than 10 ARG subtypes, respectively. Moreover, these ARGs were affiliated with more than 3 antibiotic types.

### 3.5 Environmental factors shaped airborne pathogens

**Figure 5:**
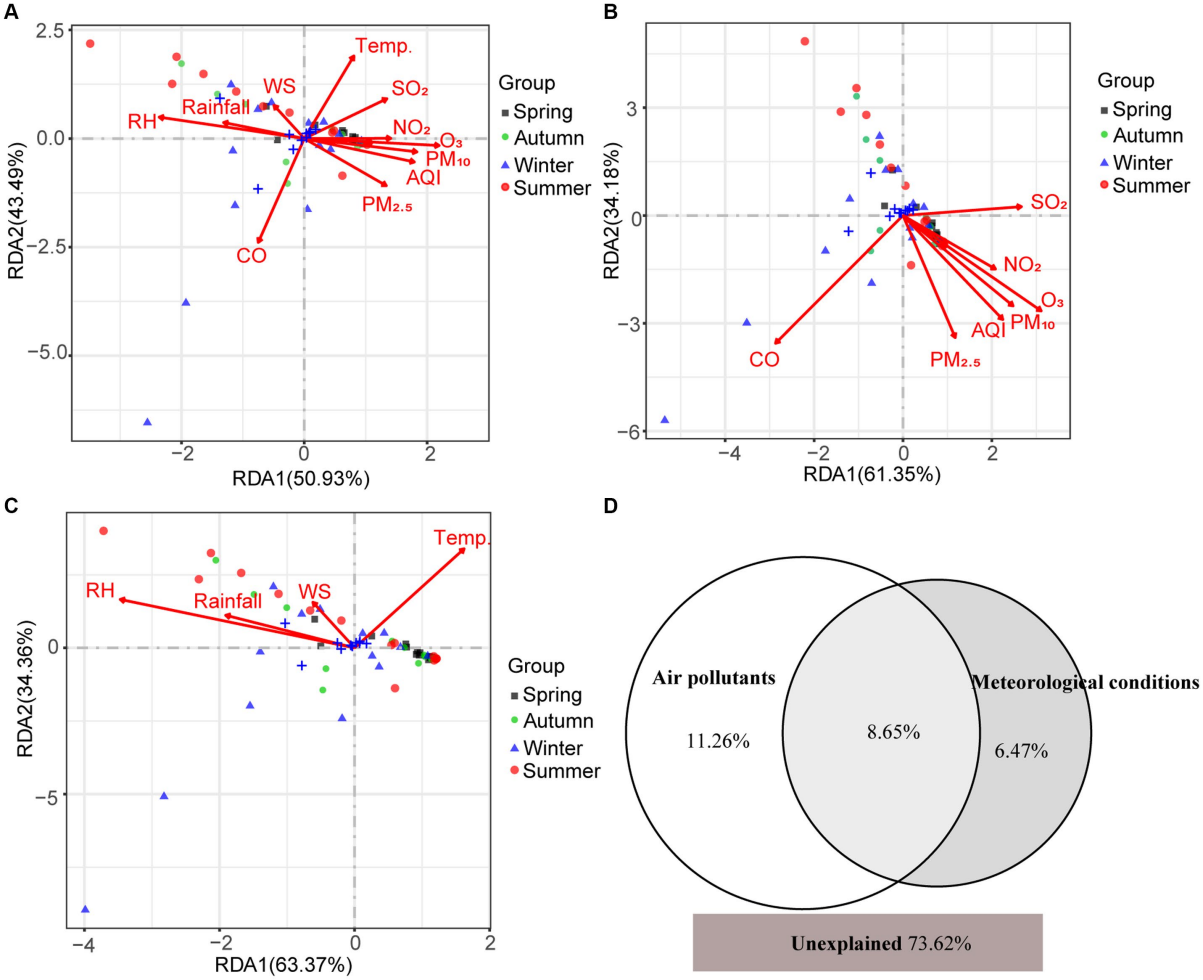
The contribution of environmental factors to variation of airborne pathogens. **(A)** RDA analysis between environmental factors and pathogen community. **(B)** RDA analysis between air pollutants and pathogen community. **(C)** RDA analysis between meteorological conditions and pathogen community. **(D)** Venn diagram for the contribution of air pollutants and meteorological conditions to variation of airborne pathogens by partial RDA. The RDA1 and RDA2 in **(A–C)** refer to the proportion of the constrained portion.

**Figure 6:**
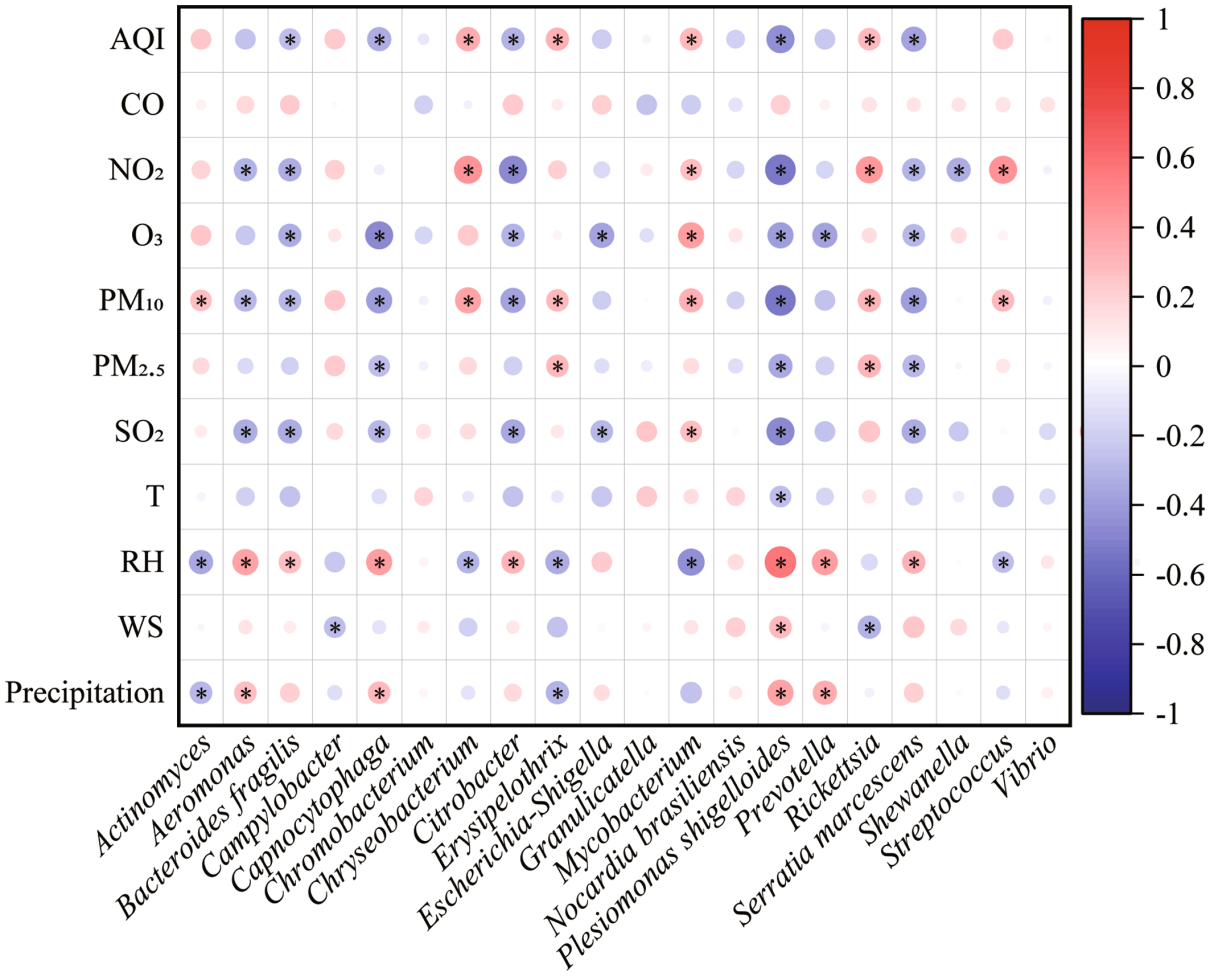
Spearman correlations of specific pathogen with environmental factors. The circle size is proportional to the spearman correlation coefficient. Red color represents positive correlation while blue color represents negative correlation. * indicates *p* < 0.05.

Mantel test showed that environmental factors shaped the pathogenic community in PM_2.5_ (*r* = 0.15, *p* = 0.01), in particular CO, O_3_, PM_2.5_, T, and RH. Both air pollutants and meteorological conditions significantly influenced the composition of pathogens (*r*_pollutants_ = 0.142, *p*_pollutants_ = 0.01; *r*_meteorology_ = 0.109, *p*_meteorology_ = 0.04). Moreover, the RDA results suggested that environmental variables totally explained 26.38% variation of pathogenic community (Figure 5). Air pollutants and meteorological conditions explained 19.91 and 15.12%, respectively. Spearman correlation analysis also found that different pathogens were influenced by environmental factors in different patterns (Figure 6). For example, meteorological conditions (relative humidity and precipitation) contributed to the diffusion of *Plesiomonas shigelloides*, *Aeromonas*, and *Bacteroides fragilis* in the air, whereas air pollutants enhanced the dissemination of *Mycobacterium*, *Streptococcus*, and *Actinomyces*.

## 4 Discussion

The seasonal characteristics of airborne microbial community have been extensively studied, and most of studies showed that air quality and meteorological conditions shaped the microbial community (Li et al., 2019; Files et al., 2020; Ruiz-Gil et al., 2020). Several human pathogens had been identified in the airborne particles (Du et al., 2018; Lu et al., 2018; Fan et al., 2019). Nevertheless, the seasonal characteristics of airborne pathogens and their potential health risk remained unclear. In this study, we found that human-to-human pathogens in winter had the highest proportion than that in other seasons (Figure 1), consistent with the result of Du et al. (2018) and Fan et al. (2019). It suggested that airborne pathogens posed greater threat to human in winter than the other seasons. Although the low temperature in winter was not conducive for microbial reproduction, high relative humidity (80.62%) favored the survival of microorganisms, which was confirmed by the high correlation between relative humidity and relative abundance of pathogens (*r* = 0.44, *p* < 0.01).

Some human pathogens such as *Prevotella*, *Escherichia-Shigella*, and *Streptococcus*, were frequently detected in the atmosphere (Lu et al., 2018; Fan et al., 2019; Jiang et al., 2022), indicating that they were resident airborne pathogens in the urban. *Prevotella* have been implicated in multiple diseases including opportunistic infections, bacterial vaginosis, oral biofilms, etc. (Tett et al., 2021). *Escherichia-Shigella* are generally regarded as proinflammatory bacteria and associated with gut dysbiosis (Li et al., 2022). *Streptococcus* are usually associated with bacteremia, acute respiratory distress syndrome, neonatal sepsis and meningitis (Hughes et al., 2009; Yacoub et al., 2014). However, part of pathogens showed seasonal preference (Figure 2 and Supplementary Figure S2), and then lead to the seasonal heterogeneity (Table 1). For instance, the dominant pathogen *Plesiomonas shigelloides* was most abundant in winter (0.44%), which also can cause intestinal diseases, sepsis and meningitis (Ingo, 2004). *Chryseobacterium* was enriched in spring (0.09%), *Prevotella* in summer (2.49%), and *Actinomyces* in autumn (0.02%), respectively. Even, *Rickettsia* was spring-specific pathogen and not detected in other seasons, which has been reported to cause many human diseases, including spotted fever, epidemic typhus, and Brill-Zinsser disease (Kelly et al., 2002; Perlman et al., 2006). Similar with the pathogen abundance, the pathogen composition also showed seasonal distribution.

Airborne microorganisms usually showed strong interactions (Fan et al., 2019; Jiang et al., 2022). In this study, we found that human pathogens in PM_2.5_ also were associated with each other (Figure 3 and Table 2), especially pathogens in summer (Supplementary Table S2). In general, significantly related pathogens may be from the same source (Pan et al., 2022). For instance, *Plesiomonas shigelloides – Aeromonas – Prevotella* had strongly positive correlation with each other in all seasons except spring, and they were widely distributed in aquatic environments (Fernández-Bravo and Figueras, 2020; Jang et al., 2023). Notably, the predominant pathogen *Plesiomonas shigelloides* was also the hub pathogen in three seasons, thus it could be used as a biomarker to monitor air pollution by pathogens.

In addition, more than half of airborne pathogens were promising hosts of high-risk ARGs (Figure 4). Moreover, some pathogens may carry multiple type of ARGs and be multi-resistant pathogens. *Plesiomonas shigelloides* had significantly correlation with ARGs belonging to beta-lactam, chloramphenicol, multidrug resistance, etc., the same as *Bacteroides fragilis*, *Escherichia-Shigella*, *Citrobacter*, and *Aeromonas*. These were consistent with previous studies (Ekundayo and Okoh, 2020; Norouzi Bazgir et al., 2020; Zhu et al., 2020; Li et al., 2021; Hassoubah, 2023). For instance, Ekundayo and Okoh (2020) have reported that *Plesiomonas shigelloides* in freshwater showed resistance to antibiotics and part of them had multi-resistance. Similarly, *Bacteroides fragilis* was previously found to be multi resistant to chloramphenicol, tetracyclines, macrolides-lincosamides-streptogramins, etc. (Niestępski et al., 2019). Li et al. (2021) found that both *Escherichia* and *Bacteroides* were the main ARG carriers in the hospital air dust. Notably, these multi-resistant pathogens tended to be more abundant in winter (Figure 2), suggesting that particulate matter in winter posed higher health risks to human.

Both air pollutants and meteorological conditions affected the seasonal distribution of pathogenic bacteria in the atmosphere (Figures 5, 6). The positive correlations between pathogens (e.g., *Mycobacterium* and *Streptococcus*) and anthropogenic activities were supported by a previous study (Fan et al., 2019). *Bacteroides fragilis*, *Prevotella*, and *Plesiomonas shigelloides* were usually found in water. This characteristic may explain their positive correlations with precipitation and relative humidity, which enhance the aerosolization of bacteria from water (Zhen et al., 2017). Therefore, the low temperature (6.92°C) and high relative humidity (80.62%) in winter promoted the enrichment of *Plesiomonas shigelloides* (Figure 2). Similarly, high PM_10_ concentration (77.36 μg/m^3^) and low relative humidity (70.95%) made *Chryseobacterium* relatively abundant in spring. Nevertheless, different from the effect of environmental factors on bacterial community (Zhen et al., 2017; Fan et al., 2019), air pollutants (19.91%) played more important role on variations of airborne pathogens than meteorological conditions (15.12%). It highlighted the effects of air quality on airborne pathogens. Overall, there were still a large proportion of variations could not be explained, indicating that some other factors might shape airborne pathogenic community. For example, some biotoxin substances absorbed on atmospheric particulate matter, such as heavy metals and volatile organic compounds, have been reported to have impacts on airborne microbial community (Li et al., 2023; Ma et al., 2023). Our findings can advance people’s knowledge of the health risks of airborne particles and manage daily production more pertinently to reduce the health risks.

This study also has some limitations. Although the reliability of PICRUSt has been validated by numerous studies using metagenomic shotgun sequencing (Zaura et al., 2015; Šamanic et al., 2021; Zhang L. et al., 2021), we applied PICRUSt to predict functional genes based on 16S rRNA gene sequencing in this study. It cannot cover the full diversity of microorganisms, and some ARGs in PM_2.5_ were extracellular. In addition, because only V3–V4 region of 16S rRNA gene was sequenced, not all pathogens can be detected at the species level. Also, a longer sampling period would be expected in the future study. Thus, the relative abundance of pathogenic bacteria may be underestimated. In the future, using metagenomic shotgun sequencing to confirm the results is necessary.

## 5 Conclusion

Airborne human-to-human pathogens in PM_2.5_ showed seasonal characteristics. The highest relative abundance of pathogens was observed in winter. Although more than half of pathogens were shared by four seasons, the pathogenic community structure shifted by season. Several pathogens tended to enrich in specific season. However, most of pathogens were obviously correlated with each other. Pathogens such as *Bacteroides fragilis*, *Aeromonas*, *Plesiomonas shigelloides* were the hub pathogens and not related with their abundances. Furthermore, these hub pathogens may be multidrug-resistant bacteria, which would pose a heavy threat on human health. Given that *Plesiomonas shigelloides* was the core pathogen in three seasons and it may be multi-resistant pathogen, it might be used as a biomarker to monitor air pollution by pathogens. Additionally, both air pollutants and meteorological conditions could partially but obviously explain the seasonal variation of pathogens.

## Data availability statement

The datasets presented in this study can be found in online repositories. The names of the repository/repositories and accession number(s) can be found in the article/Supplementary material.

## Author contributions

ZZ: Data curation, Formal analysis, Writing – original draft. YP: Funding acquisition, Investigation, Supervision, Writing – review & editing. XH: Data curation, Investigation, Writing – review & editing. TY: Writing – review & editing, Funding acquisition. CL: Writing – review & editing. XC: Writing – review & editing. ZX: Writing – review & editing, Formal analysis, Methodology, Supervision.

## Footnotes

^1^http://www.nhc.gov.cn/qjjys/s7948/202308/b6b51d792d394fbea175e4c8094dc87e.shtml

^2^https://www.genome.jp/kegg/annotation/br01600.html
