# Supplementary material for: Seasonal distribution of human-to-human pathogens in airborne PM2.5 and their potential high-risk ARGs
Source: Front Microbiol. 2024 Jul 4;15:1422637. doi: 10.3389/fmicb.2024.1422637 (PMC11254772; doi:10.3389/fmicb.2024.1422637)

*Supplemental materials of*

**Seasonal distribution of human-to-human pathogens in airborne PM_2.5_ and their potential high-risk ARGs**

Zhiwei Zuo^1^, Yuanyuan Pan^1*^, Xueyun Huang^1^, Tao Yuan^1^, Cheng Liu^1^, Xihong Cai^1^, Zhongji Xu^2*^

1. Jiangxi Provincial Key Laboratory of Genesis and Remediation of Groundwater Pollution, East China University of Technology, Nanchang 330013, China

2. Jiangxi Center for Patriotic Health and Health Promotion, Nanchang, 330013, China

Corresponding author: Yuanyuan Pan

E-mail: panyyuan@ecut.edu.cn

**Table S1** The raw data information for samples used in this study.

| Season | Bioproject ID | Biosample ID | Samples |
| --- | --- | --- | --- |
| Spring | PRJNA867037 | SRR22387535 | S1 |
|  |  | SRR22387534 | S2 |
|  |  | SRR22387533 | S3 |
|  |  | SRR22387532 | S4 |
|  |  | SRR22387531 | S5 |
|  |  | SRR22387530 | S6 |
|  |  | SRR22387528 | S7 |
|  |  | SRR22387527 | S8 |
|  |  | SRR22387526 | S9 |
|  |  | SRR22387525 | S10 |
|  |  | SRR22387524 | S11 |
|  |  | SRR22387523 | S12 |
|  |  | SRR22387522 | S13 |
|  |  | SRR22387521 | S14 |
| Summer | PRJNA867124 | SAMN30184902 | Su1 |
|  |  | SAMN30184903 | Su2 |
|  |  | SAMN30184904 | Su3 |
|  |  | SAMN30184905 | Su4 |
|  |  | SAMN30184906 | Su5 |
|  |  | SAMN30184907 | Su6 |
|  |  | SAMN30184908 | Su7 |
|  |  | SAMN30184909 | Su8 |
|  |  | SAMN30184910 | Su9 |
|  |  | SAMN30184911 | Su10 |
|  |  | SAMN30184912 | Su11 |
|  |  | SAMN30184913 | Su12 |
|  |  | SAMN30184914 | Su13 |
|  |  | SAMN30184915 | Su14 |
|  |  | SAMN30184916 | Su15 |
| Autumn | PRJNA867124 | SAMN30184845 | G1 |
|  |  | SAMN30184846 | G2 |
|  |  | SAMN30184847 | G3 |
|  |  | SAMN30184848 | G4 |
|  |  | SAMN30184849 | G7 |
|  |  | SAMN30184850 | G8 |
|  |  | SAMN30184851 | G9 |
|  |  | SAMN30184852 | G10 |
|  |  | SAMN30184853 | G11 |
|  |  | SAMN30184854 | G12 |
|  |  | SAMN30184855 | G13 |
|  |  | SAMN30184856 | G14 |
|  |  | SAMN30184857 | G15 |
|  |  | SAMN30184858 | G16 |
| Winter | PRJNA867124 | SAMN30184872 | N3 |
|  |  | SAMN30184873 | N4 |
|  |  | SAMN30184874 | N5 |
|  |  | SAMN30184875 | N6 |
|  |  | SAMN30184876 | N7 |
|  |  | SAMN30184877 | N8 |
|  |  | SAMN30184878 | N9 |
|  |  | SAMN30184879 | N10 |
|  |  | SAMN30184880 | N11 |
|  |  | SAMN30184881 | N12 |
|  |  | SAMN30184882 | N13 |
|  |  | SAMN30184883 | N14 |
|  |  | SAMN30184884 | N15 |
|  |  | SAMN30184885 | N16 |

**Table S2** The Spearman correlation coefficient (r) and p value between pathogen and pathogen in the co-occurrence network.

| **Spring** | Source | Target | r | p value |
| --- | --- | --- | --- | --- |
|  | *Capnocytophaga* | *Chryseobacterium* | -0.563 | 0.036 |
|  | *Bacteroides fragilis* | *Campylobacter* | 0.796 | 0.036 |
|  | *Chryseobacterium* | *Escherichia-Shigella* | 0.587 | 0.027 |
|  |  |  |  |  |
| **Summer** | Source | Target | r | p value |
|  | *Chromobacterium* | *Campylobacter* | 0.732 | 0.002 |
|  | *Chromobacterium* | *Erysipelothrix* | 1 | 0 |
|  | *Campylobacter* | *Erysipelothrix* | 0.732 | 0.002 |
|  | *Campylobacter* | *Shewanella* | 0.561 | 0.030 |
|  | *Campylobacter* | *Bacteroides fragilis* | 0.596 | 0.019 |
|  | *Campylobacter* | *Escherichia-Shigella* | 0.593 | 0.020 |
|  | *Nocardia brasiliensis* | *Mycobacterium* | 0.669 | 0.006 |
|  | *Capnocytophaga* | *Aeromonas* | 0.599 | 0.018 |
|  | *Capnocytophaga* | *Plesiomonas shigelloides* | 0.631 | 0.012 |
|  | *Shewanella* | *Bacteroides fragilis* | 0.516 | 0.049 |
|  | *Vibrio* | *Serratia marcescens* | 0.640 | 0.010 |
|  | *Vibrio* | *Aeromonas* | 0.750 | 0.001 |
|  | *Vibrio* | *Citrobacter* | 0.543 | 0.036 |
|  | *Vibrio* | *Bacteroides fragilis* | 0.594 | 0.019 |
|  | *Vibrio* | *Mycobacterium* | -0.617 | 0.014 |
|  | *Vibrio* | *Streptococcus* | 0.618 | 0.014 |
|  | *Vibrio* | *Plesiomonas shigelloides* | 0.713 | 0.003 |
|  | *Serratia marcescens* | *Aeromonas* | 0.571 | 0.026 |
|  | *Serratia marcescens* | *Bacteroides fragilis* | 0.774 | 0.001 |
|  | *Serratia marcescens* | *Streptococcus* | 0.596 | 0.019 |
|  | *Serratia marcescens* | *Plesiomonas shigelloides* | 0.645 | 0.009 |
|  | *Serratia marcescens* | *Escherichia-Shigella* | 0.655 | 0.008 |
|  | *Aeromonas* | *Citrobacter* | 0.627 | 0.012 |
|  | *Aeromonas* | *Bacteroides fragilis* | 0.564 | 0.028 |
|  | *Aeromonas* | *Mycobacterium* | -0.557 | 0.031 |
|  | *Aeromonas* | *Streptococcus* | 0.739 | 0.002 |
|  | *Aeromonas* | *Plesiomonas shigelloides* | 0.930 | 0 |
|  | *Aeromonas* | *Prevotella* | 0.684 | 0.005 |
|  | *Chryseobacterium* | *Plesiomonas shigelloides* | 0.539 | 0.038 |
|  | *Citrobacter* | *Bacteroides fragilis* | 0.537 | 0.039 |
|  | *Citrobacter* | *Mycobacterium* | -0.741 | 0.002 |
|  | *Citrobacter* | *Streptococcus* | 0.721 | 0.002 |
|  | *Citrobacter* | *Plesiomonas shigelloides* | 0.701 | 0.004 |
|  | *Citrobacter* | *Prevotella* | 0.745 | 0.001 |
|  | *Bacteroides fragilis* | *Streptococcus* | 0.807 | 0.000 |
|  | *Bacteroides fragilis* | *Plesiomonas shigelloides* | 0.636 | 0.011 |
|  | *Bacteroides fragilis* | *Escherichia-Shigella* | 0.792 | 0.000 |
|  | *Bacteroides fragilis* | *Prevotella* | 0.631 | 0.012 |
|  | *Mycobacterium* | *Streptococcus* | -0.671 | 0.006 |
|  | *Mycobacterium* | *Plesiomonas shigelloides* | -0.623 | 0.013 |
|  | *Mycobacterium* | *Prevotella* | -0.591 | 0.020 |
|  | *Streptococcus* | *Plesiomonas shigelloides* | 0.730 | 0.002 |
|  | *Streptococcus* | *Escherichia-Shigella* | 0.670 | 0.006 |
|  | *Streptococcus* | *Prevotella* | 0.937 | 0.000 |
|  | *Plesiomonas shigelloides* | *Prevotella* | 0.686 | 0.005 |
|  | *Escherichia-Shigella* | *Prevotella* | 0.600 | 0.018 |
|  |  |  |  |  |
| **Autumn** | Source | Target | r | p value |
|  | *Campylobacter* | *Erysipelothrix* | 0.734 | 0.003 |
|  | *Erysipelothrix* | *Aeromonas* | -0.548 | 0.042 |
|  | *Erysipelothrix* | *Plesiomonas shigelloides* | -0.609 | 0.021 |
|  | *Capnocytophaga* | *Vibrio* | 0.593 | 0.026 |
|  | *Capnocytophaga* | *Mycobacterium* | -0.609 | 0.021 |
|  | *Vibrio* | *Actinomyces* | 0.710 | 0.004 |
|  | *Vibrio* | *Aeromonas* | 0.648 | 0.012 |
|  | *Actinomyces* | *Aeromonas* | 0.631 | 0.016 |
|  | *Aeromonas* | *Bacteroides fragilis* | 0.442 | 0.050 |
|  | *Aeromonas* | *Mycobacterium* | 0.533 | 0.007 |
|  | *Aeromonas* | *Plesiomonas shigelloides* | 0.868 | 0.000 |
|  | *Aeromonas* | *Escherichia-Shigella* | 0.560 | 0.037 |
|  | *Aeromonas* | *Prevotella* | 0.640 | 0.014 |
|  | *Citrobacter* | *Plesiomonas shigelloides* | 0.578 | 0.030 |
|  | *Citrobacter* | *Escherichia-Shigella* | 0.622 | 0.018 |
|  | *Citrobacter* | *Prevotella* | 0.657 | 0.011 |
|  | *Bacteroides fragilis* | *Plesiomonas shigelloides* | 0.542 | 0.045 |
|  | *Bacteroides fragilis* | *Escherichia-Shigella* | 0.807 | 0.000 |
|  | *Bacteroides fragilis* | *Prevotella* | 0.668 | 0.009 |
|  | *Bacteroides fragilis* | *Mycobacterium* | -0.735 | 0.003 |
|  | *Mycobacterium* | *Plesiomonas shigelloides* | -0.763 | 0.002 |
|  | *Mycobacterium* | *Escherichia-Shigella* | -0.582 | 0.029 |
|  | *Mycobacterium* | *Prevotella* | -0.793 | 0.001 |
|  | *Plesiomonas shigelloides* | *Escherichia-Shigella* | 0.596 | 0.025 |
|  | *Plesiomonas shigelloides* | *Prevotella* | 0.881 | 0.000 |
|  | *Escherichia-Shigella* | *Prevotella* | 0.727 | 0.003 |
|  |  |  |  |  |
| **Winter** | Source | Target | r | p value |
|  | *Erysipelothrix* | *Vibrio* | -0.696 | 0.006 |
|  | *Erysipelothrix* | *Serratia marcescens* | -0.537 | 0.048 |
|  | *Erysipelothrix* | *Actinomyces* | 0.599 | 0.024 |
|  | *Erysipelothrix* | *Aeromonas* | -0.663 | 0.010 |
|  | *Erysipelothrix* | *Bacteroides fragilis* | -0.713 | 0.004 |
|  | *Erysipelothrix* | *Mycobacterium* | 0.600 | 0.023 |
|  | *Erysipelothrix* | *Plesiomonas shigelloides* | -0.717 | 0.004 |
|  | *Capnocytophaga* | *Aeromonas* | 0.558 | 0.038 |
|  | *Capnocytophaga* | *Prevotella* | 0.717 | 0.004 |
|  | *Shewanella* | *Chryseobacterium* | -0.627 | 0.017 |
|  | *Vibrio* | *Plesiomonas shigelloides* | 0.579 | 0.030 |
|  | *Actinomyces* | *Mycobacterium* | 0.698 | 0.006 |
|  | *Actinomyces* | *Plesiomonas shigelloides* | -0.605 | 0.022 |
|  | *Aeromonas* | *Plesiomonas shigelloides* | 0.704 | 0.005 |
|  | *Aeromonas* | *Prevotella* | 0.659 | 0.010 |
|  | *Chryseobacterium* | *Streptococcus* | 0.722 | 0.004 |
|  | *Citrobacter* | *Bacteroides fragilis* | 0.638 | 0.014 |
|  | *Citrobacter* | *Escherichia-Shigella* | 0.598 | 0.024 |
|  | *Bacteroides fragilis* | *Mycobacterium* | -0.727 | 0.003 |
|  | *Bacteroides fragilis* | *Escherichia-Shigella* | 0.711 | 0.004 |
|  | *Mycobacterium* | *Streptococcus* | 0.550 | 0.042 |
|  | *Plesiomonas shigelloides* | *Prevotella* | 0.582 | 0.029 |

**Fig. S1** Shannon rarefactions to evaluate the sequencing depth.


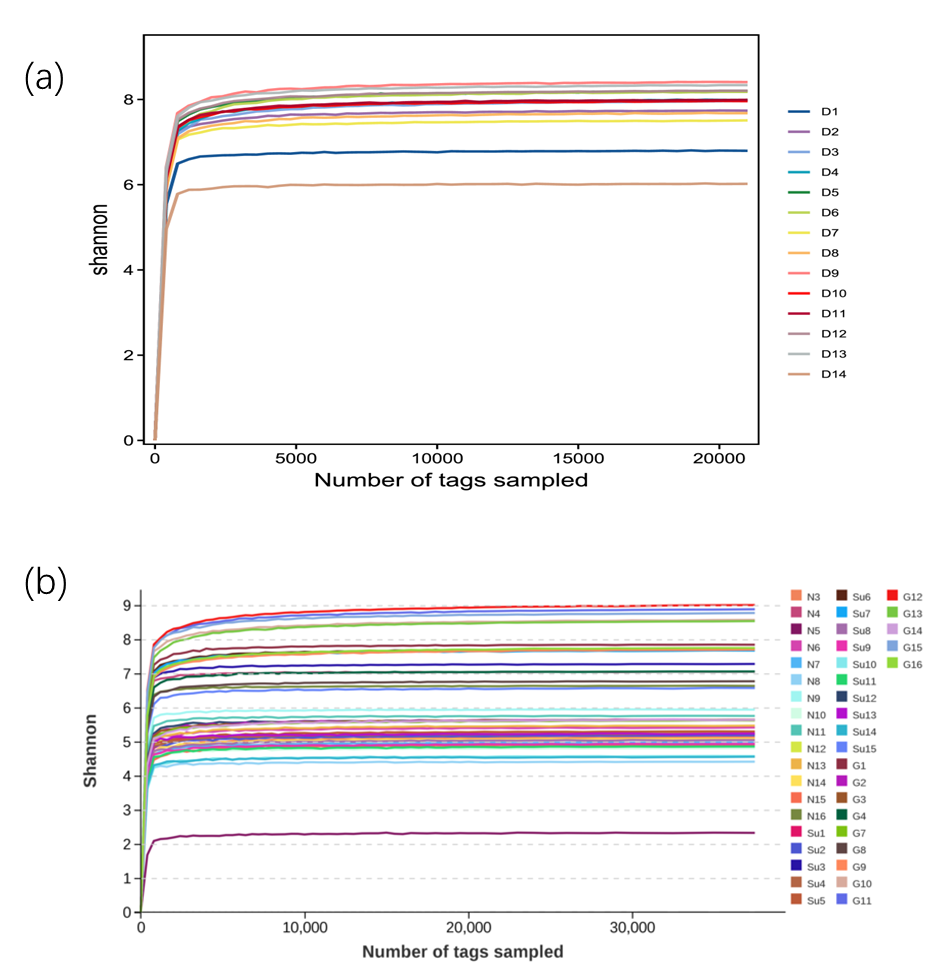


**Fig. S2** Heatmap based on the relative abundance of pathogens in each season. The row was standardized to show the enriched pathogens in each season.


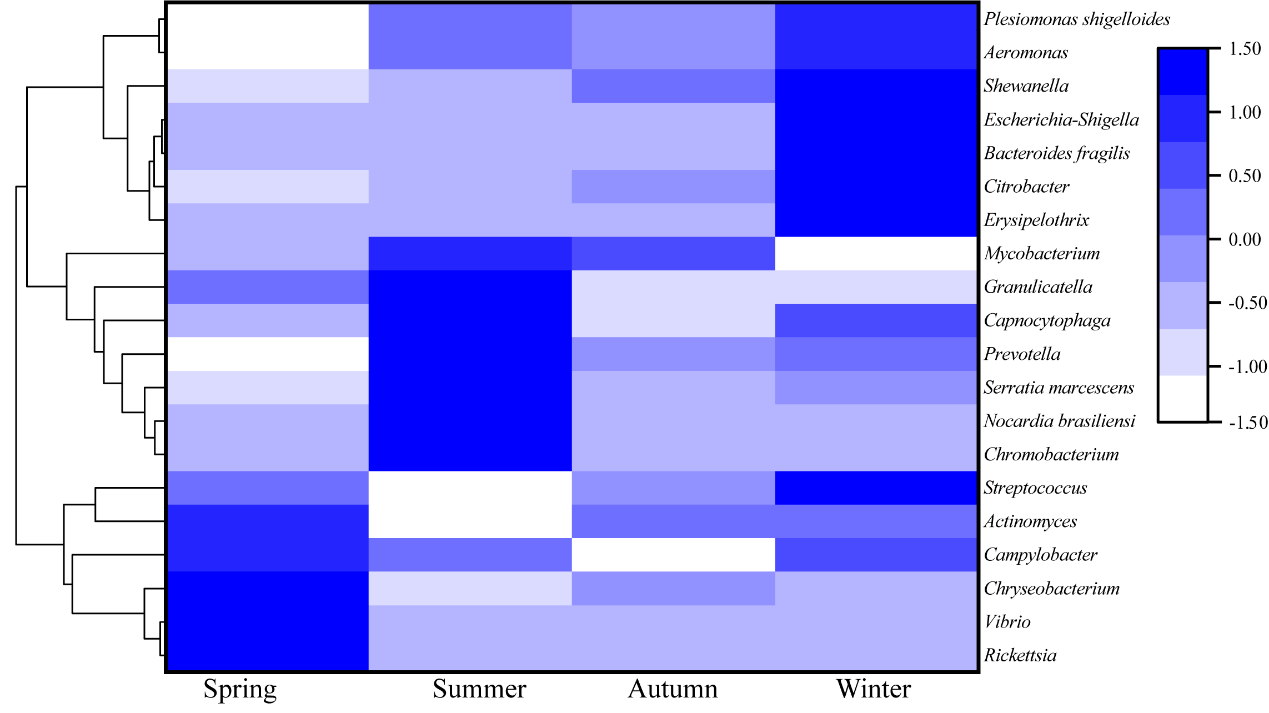

Supplement: Supplementary file 1 [file Data_Sheet_1.docx]
